# Epidemiological and Molecular Investigations on *Salmonella* Responsible for Gastrointestinal Infections in the Southwest of Shanghai From 1998 to 2017

**DOI:** 10.3389/fmicb.2019.02025

**Published:** 2019-09-18

**Authors:** Xulin Qi, Pei Li, Xiaogang Xu, Yiqun Yuan, Shurui Bu, Dongfang Lin

**Affiliations:** ^1^Department of Infection, Jinshan Hospital Affiliated to Fudan University, Shanghai, China; ^2^Institute of Antibiotics, Huashan Hospital Affiliated to Fudan University, Shanghai, China

**Keywords:** diarrhea, *Salmonella* strains, food poisoning, epidemiology, PFGE, Chinese patients

## Abstract

**Purpose:**

To investigate the characteristics of gastrointestinal infections in Southwest Shanghai.

**Methods:**

Clinical and epidemiological characteristics of patients with *Salmonella* infections between 1998 and 2017 admitted to the Jinshan Hospital in the Southwest of Shanghai were retrospectively analyzed. A total of 565 isolated *Salmonella* strains were classified by serotyping and pulsed field gel electrophoresis (PFGE).

**Results:**

From 1998 to 2006, diarrhea was mainly caused by *Vibrio parahaemolyticus* followed by *Shigella* and *Salmonella*. From 2007 to 2010, *Vibrio parahaemolyticus* infection was the major cause of diarrhea followed by *Salmonella* and *Shigella*. From 2011 to 2017, *Salmonella* infections became the main cause of diarrhea after *Vibrio parahaemolyticus*. *Salmonella* infections increased from 2006 on and peaked between May and October, accounting for 82.48% of yearly infections. Patients with *Salmonella* infections (90.5%) had a history of eating unclean food, abdominal pain (58.05%), diarrhea ≥5 times a day (50.44%), moderate fever (24.96%) and increased fecal leukocytes (41.42%). From 1998 to 2017, infected specimens from clinical cases were dominated by *Salmonella enterica* serovar Typhimurium (*S*. Typhimurium) (21.59%) followed by *Salmonella enterica* serovar Enteritis (*S*. Enteritidis) (16.81%), *Salmonella enterica* serotype London (6.55%) and *Salmonella* group B (13.10%). Other species included *Salmonella enterica* serovar Thompson, *Salmonella enterica* serovar Saintpaul, *Salmonella* group D, *Salmonella* group C, *Salmonella enterica* serovar Choleraesuis and *Salmonella enterica* serovar Aberdeen. The PFGE classification of *Salmonella* serovars in 2008–2017 demonstrated that *S*. Enteritidis had 9 PFGE banding patterns and *S*. Typhimurium 16 with varying degrees of similarity among *S*. Enteritidis and *S*. Typhimurium. The results of antibiotic susceptibility tests for the 330 *Salmonella* strains revealed that fosfomycin had the highest sensitivity rate (97.5%) followed by levofloxacin and ceftriaxone (81%), and ampicillin/sulbactam (78.2%). The resistance to piperacillin and ciprofloxacin was 60.9 and 50.61%, respectively.

**Conclusion:**

The features of onset, epidemiological characteristics and molecular subtyping of *Salmonella* were conducive to clinical diagnosis, rational use of antibiotics and improved therapeutic efficacy.

## Introduction

Infectious diarrhea is a common and frequently occurring disease worldwide. The WHO reported that the incidence rate is ranked second compared to all infectious diseases (Lamberti et al., 2014). The pathogens that cause infectious diarrhea are widespread and include viruses, bacteria, fungi and parasites. The bacterial pathogens commonly involved include *Shigella*, *Salmonella*, enteropathogenic *Escherichia coli* (EPEC), *Vibrio parahaemolyticus* and other organisms (Evyline Isingoma et al., 2018).

*Salmonella* is an important pathogen that poses a significant threat to human health, with more than 2,600 species of serotypes discovered to date (Bugarel et al., 2018). *Salmonella* infection is an infectious intestinal disease causing symptoms that include enteric typhoid-like and paratyphoid-like fever, gastroenteritis and various forms of extra-intestinal inflammation such as bacteremia, cholecystitis, and pyelonephritis (Brenner et al., 2000; Fàbrega and Vila, 2013). Moreover, the virulence of different *Salmonella* types can be quite different. For example, *Salmonella enterica* serovar Anatis presents as an asymptomatic phenotype, while septicemia caused by *Salmonella enterica* serovar Choleraesuis may lead to death (Guo et al., 2018). *S*. Typhimurium produces the symptoms of dysentery and *S*. Enteritidis often causes gastroenteritis (Kurtz et al., 2017). Therefore, *Salmonella* infections produce digestive symptoms among other general ailments including fever. Furthermore, there are many strain types, which makes it difficult to diagnose rapidly and accurately the condition (Bugarel et al., 2018). In a previous study conducted in 28 sentinel hospitals located in five geographic regions of the Henan Province six different main serotypes were detected (Xia et al., 2009). Similar results from studies in Africa reported infections in patients with co-morbidities affecting the immune system, the predominance of *S*. Typhimurium and other *Salmonella* serovars, and the presence of drug-resistance in isolates (Albert et al., 2019).

Therefore, in order to establish unequivocally the epidemiological characteristics and clinical features of *Salmonella* in Southwest of Shanghai, and to provide assistance with judging the condition, diagnosis and treatment options, 20 years worth of cases of *Salmonella* intestinal infections in our hospital were collected to analyze their clinical features and epidemiological characteristics.

Pulsed field gel electrophoresis (PFGE) can be used to isolate long linear DNA sequences, with banding patterns appearing on the agarose gel based on the different lengths of chromosome segments. PFGE has the highest discriminatory power among the different molecular typing methods for the investigation of clonal relationships between bacteria (Ersoy Omeroglu, 2015). Therefore, we used PFGE for homogenous bacterial typing, which directly or indirectly reflected the variation and differentiation of the pathogens studied. In particular, PFGE was used to analyze changes in *Salmonella* intestinal infections, using fecal routine examinations and feces culture investigations. The cases of positive *Salmonella* infections in Shanghai were analyzed and classified.

## Patients and Methods

### Materials and Methods

#### Inclusion Criteria

The study was a retrospective study, but was conducted in accordance with good clinical practice, the Principles of the Declaration of Helsinki and the requirements of the local hospital (Jinshan Hospital) ethics committee, which waived informed consent. Between 1998 and 2017, patients who visited the Enteric Disease Clinic of Jinshan Hospital with two or more of the following symptoms were selected for inclusion in the study. The inclusion criteria were: diarrhea 3 or more times in 24 h with abnormal feces; fever with a temperature ≥38°C accompanied by headache, chills and general fatigue; vomiting, diarrhea, abdominal pain, and abnormal feces such as diluted feces, water-like feces, mucus-like feces and bloody feces. Patients who exhibited at least two of the symptoms described above were included.

##### Inclusion

This study analyzed all demographic data and the clinical performance records of all enrolled patients. Pathogenic *Salmonella*-positive cases and laboratory data were also analyzed.

##### Exclusion criteria

From 1998 to 2017, the same strain from the same location in the same patient was excluded from all types of bacterial culture specimens examined.

##### Fecal sampling method

Patients were first informed of the purpose of fecal culture and then advised to defecate in a clean toilet bowl after emptying their bladders. A sterile bamboo stick was used to obtain a small amount of excrement from the central part or from purulent blood or mucus, which was then placed in a sterile culture bottle. The specimen was then sent for detailed laboratory analysis. The collection of all stool specimens was performed in accordance with the Institutional Review Board guidelines of Jinshan Hospital.

### Isolation, Culture and Identification of Bacteria

#### Bacterial Isolation

Freshly collected fecal samples were preserved in Cary-Blair transport medium and inoculated on SS (for selective separation of *Shigella* and *Salmonella*) and TCBS plates (thiosulfate citrate bile salts-sucrose agar culture medium) for selective separation of *Vibrio parahaemolyticus*). After 6–10 h of culture at 35°C, suspicious colonies were selected for preliminary biochemical identification using the serum agglutination method and then further cultured to obtain a pure strain.

#### Bacteriological Examination

An automatic bacteria identification instrument (VITEK 2 Compact, Biomérieux, France) was used to identify pathogenic bacteria from pure cultured strains.

#### Antimicrobial Susceptibility Test Method

Minimum inhibitory concentrations (MICs) were determined using custom dehydrated MicroScan broth microdilution (Siemens Medical Solutions Diagnostics), following Clinical and Laboratory Standards Institute (CLSI) guidelines (Cockerill, 2012) and relative susceptibility interpretations were based on CLSI clinical breakpoints (CLSI, 2017).

Thirteen antimicrobial agents commonly used to treat diarrhea were tested. Results were only included in the analysis when corresponding quality control isolate test results were in accordance with CLSI, 2017 guidelines, which were unchanged between 2014 and 2017 and therefore within an acceptable range.

#### PFGE Subtype Method

*Salmonella* strains were classified according to a previous publication (Ribot et al., 2002). A standard *Salmonella* strain H9812 was used as the molecular weight control marker. The prepared DNA plugs with *Salmonella* strains were digested with *Xba*I for 3 h at 37°C. The electrophoresis conditions were: voltage 6 V/cm; pulse duration 2.2 s to 63.8 s; linear transformation with a convert angle of 120°; electrophoresis period and temperature 19 h at 14°C, respectively, which was a modified method from the report of Laconcha et al. (2000). In a PFGE electrophoretogram, each lane represents one isolated strain. With the effect of endonuclease, different strains present as different numbers and band sizes. After calibration by a unified molecular weight standard based on the H9812 strain, the strains which showed a high degree of resemblance over 60% were classified as identical PFGE types.

### Statistical Methods

Data was analyzed by using SPSS Statistics for Windows (version 10.0, SPSS Inc., United States). A *t*-test was used for continuous quantitative data that was normally distributed and a chi-squared test to compare qualitative data. Results of PFGE were processed through BioNumerics software version 3.3. Clustering analysis was based on the unweighted pair group method using arithmetic averages (UPGMA). The dice coefficient, based on bands comparison, was used to determine the similarity of PFGE bands. Those in which the homology was over 60% were considered to be the same PFGE type.

## Results

### Patients’ Basic Information

In total, 32,544 patients with acute diarrhea as the first symptom, who were admitted to our enteric disease clinic located in the Southwest of Shanghai between 1998 and 2017, were retrospectively analyzed. Most of the patients came from Shanghai, but some were from the neighbor city Jiaxing. A total of 29,589 case specimens were sent for laboratory examination, which represented an inspection rate of circa 90%. A total of 2,849 pathogen-positive cases were collected and the bacterial isolation rate was 9.63%.

From 1998 to 2010, *Vibrio parahaemolyticus* infection (59.49–99.5%) was the major cause of acute diarrhea in Southwest Shanghai, followed by *Salmonella* (0.00–37.97%) and to a lesser extent *Shigella* infection (0.0–11.8%). However, significant changes occurred between 2011 and 2017. The *Salmonella* infection rate became the number one cause of diarrhea (53.13–85.00%) followed by *Vibrio parahaemolyticus* (15.00–46.88%) with almost no infections due to *Shigella*; only 13 strains of *Shigella* were found in 2011. In recent years, the age range of patients with *Salmonella* infection as the major infective pathogen was 6 months to 83 years (average 35.55 ± 12.82 years). The isolation rate of positive patients with pathogenic *Salmonella* infection between 1998 and 2017 was 0∼85.00%. From 1998 to 2006 <10 strains were isolated each year, with the total incidence being 0–0.90%. From 2007 to 2015, isolated strains began to increase (18–58 strains) with the total incidence rate reaching 23.08% to 83.87%. Between 2016 and 2017, 102 and 120 strains were isolated with an 85.0 and 75.47% incidence rate, respectively. It is worth noting that the incidence rate of *Shigella* infection in Southwest Shanghai from 2012 was very low and practically zero, while *Salmonella* infections became the dominant infectious pathogens (Supplementary Table S1).

### Correlation Between Infections and Time

**FIGURE 1:**
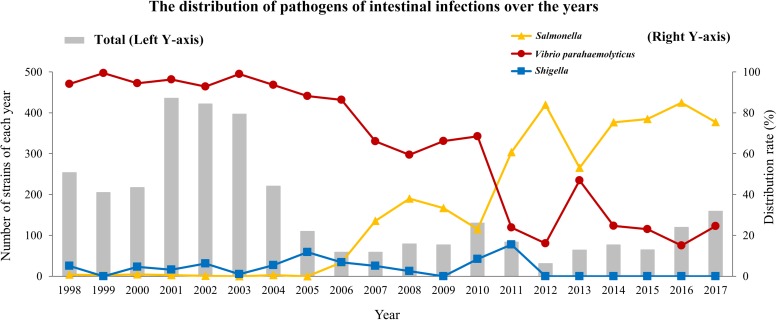
Distribution of *Salmonella*, *Vibrio parahaemolyticus* and *Shigella* caused intestinal infections between 1998 and 2017.

Figure 1 shows a histogram of the pathogens that caused intestinal infections between 1998 and 2017.

**FIGURE 2:**
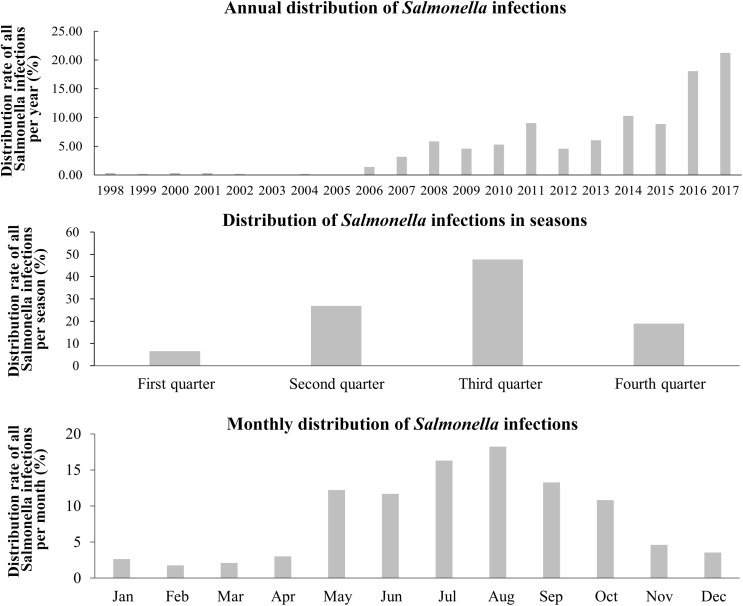
Yearly, seasonally and monthly distribution of *Salmonella* infections in the Southwest of Shanghai from 1998 to 2017.

In terms of years, *Salmonella* infections began to increase from 2006 and were mainly centralized in the third quarter, and then in the second and fourth quarters of each year. From the monthly distribution, May to October was the peak period of *Salmonella* infections, accounting for 82.48% but it should be noted that infections still occurred throughout the year (Figure 2).

### Clinical Characteristics of *Salmonella* Infection

Among 565 patients with *Salmonella* infection, 58.05% had abdominal pain, 50.44% diarrhea >5 times daily and 24.96% moderate fever with an average oral temperature of 38.7 ± 0.7°C. Patients with upper gastrointestinal symptoms accompanied by nausea and emesis accounted for 30.97 and 18.05% of cases, respectively. A total of 41.42% of patients had increasing fecal leukocyte counts and 90.5% mentioned they had consumed unclean food. Normally after having eaten unclean food, the minimum time to onset of symptoms was 1 h and the maximum time 72 h (Table 1 and Supplementary File S1).

**TABLE 1:** Clinical characteristics of *Salmonella* infections in the southwest of Shanghai in 1998 to 2017.

Clinical symptoms	*Salmonella* infections (565 cases)	
	*n*	Percentage (%)
Fever	141	24.96
Abdominal pain	328	58.05
Nausea	175	30.97
Emesis	102	18.05
Tenesmus	0	0
Dehydration	13	2.30
Defecating frequency >5 times	285	50.44
Defecating frequency ≤5 times	281	49.73
Fecal leukocyte increase	234	41.42
Fecal red blood cell increase	108	19.12

### Results of Serotyping

**FIGURE 3:**
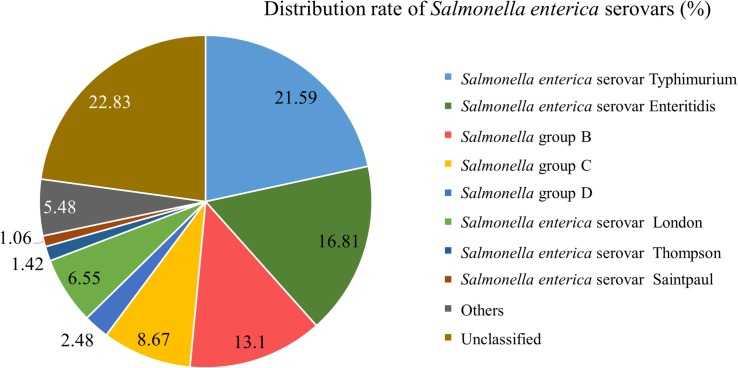
Distribution ratio of different serovars of *Salmonella enterica* in the Southwest of Shanghai from 1998 to 2017.

From 1998 to 2017, infected specimens from clinical cases in Southwest Shanghai were dominated by *S*. Typhimurium (21.59%), followed by *S*. Enteritidis (16.81%), *Salmonella enterica* serotype London (6.55%) and *Salmonella* group B (13.10%) (Figure 3). Other species included *Salmonella enterica* serovar Thompson, *Salmonella enterica* serovar Saintpaul, *Salmonella* group D, *Salmonella* group C, *Salmonella enterica* serovar Choleraesuis and *Salmonella enterica* serovar Aberdeen.

### The Susceptibility of Common Antimicrobial Agents to *Salmonella*

**FIGURE 4:**
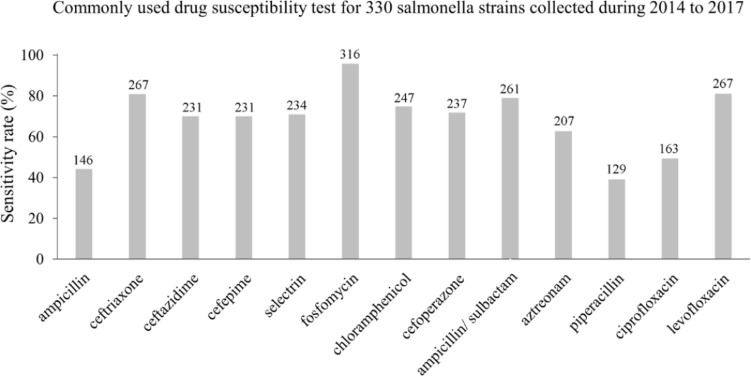
The susceptibilities of *Salmonella* infections to 13 antibiotics in the Southwest of Shanghai between 2014 and 2017.

Commonly used antibiotics susceptibility tests for the 330 *Salmonella* strains collected between 2014 and 2017 revealed that fosfomycin had the highest sensitivity rate, reaching 97.5%, followed by levofloxacin and ceftriaxone (81%) and ampicillin/sulbactam (78.2%) (Figure 4). The sensitivity rates of piperacillin and ciprofloxacin were 39.1 and 49.39%, decreasing by 30.26 and 3.96%, respectively, results comparable to previous studies (Demirtas et al., 2012; Shubert et al., 2014; Figure 4).

#### PFGE Subtypes of *Salmonella S*. Enteritidis and *Salmonella S*. Typhimurium From 2014 to 2017

**FIGURE 5:**
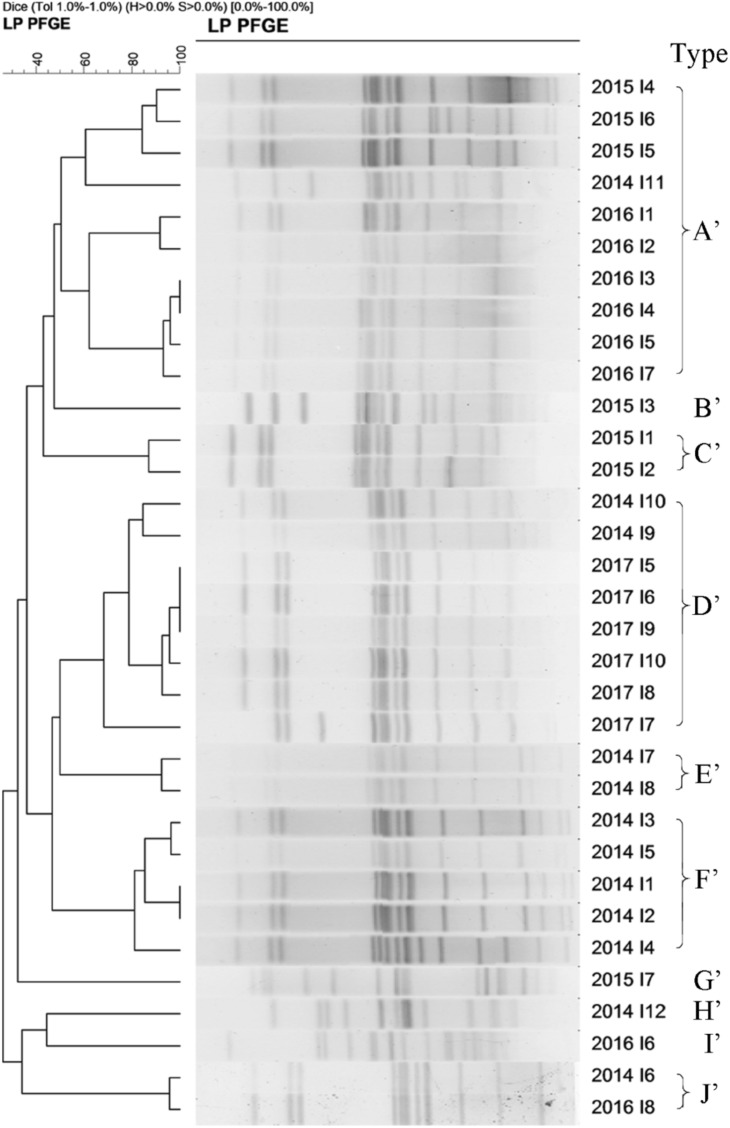
The 33 *S.* Enteritidis strains collected from 2014 to 2017 were classify into 9 types (named **A’–J’**) based on a >60% similarity.

**FIGURE 6:**
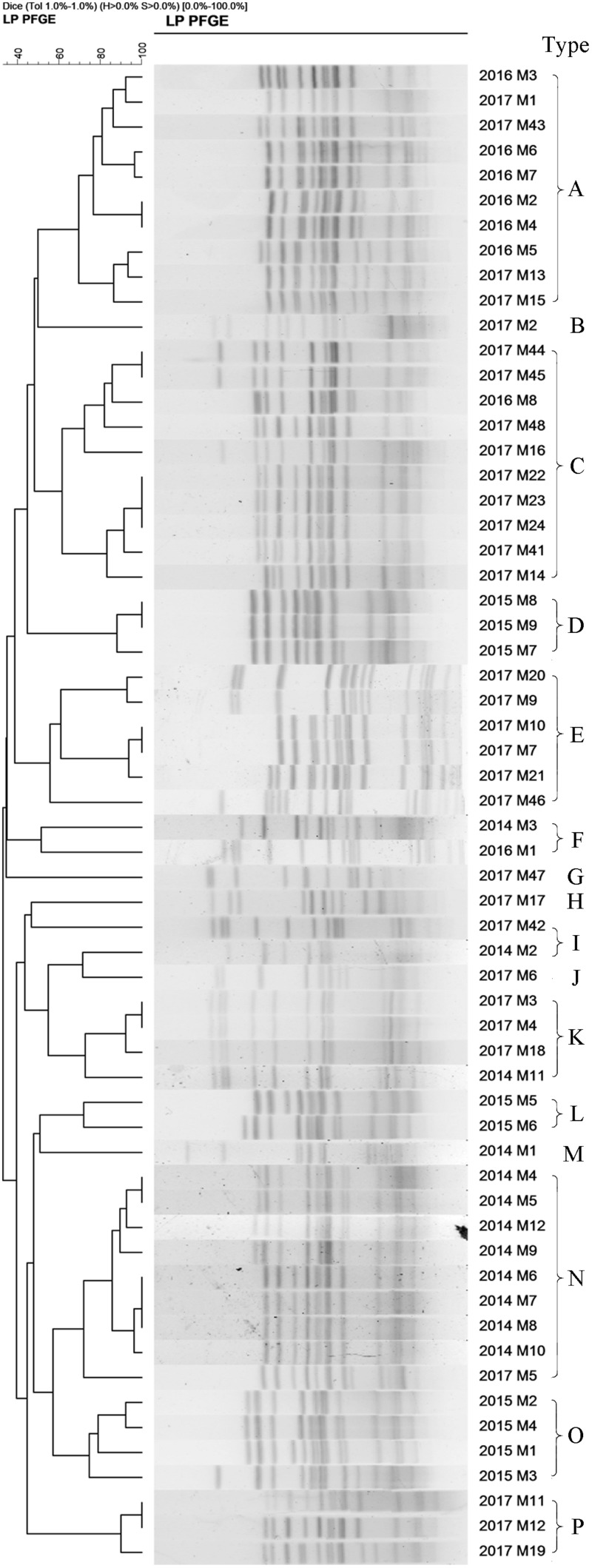
The 60 *S*. Typhimurium strains collected from 2014 to 2017 were classify into 16 types **(A–P)** based on a >60% similarity (*Endonuclease Xba*I).

A total of 33 *S.* Enteritidis strains and 60 *S*. Typhimurium strains were collected from 2014–2017 and digested with *Xba*I enzyme and analyzed using PFGE. The results showed that the DNA bands had a good separation. The size of the bands ranged from 20.5 to 1,135 kb and the number of bands from 12 to 16. Cluster analysis, using the BioNumerics module, classified *S*. Enteritidis into 9 PFGE types (named A’–J’) and *S.* Typhimurium into 16 types (named A–P) based on a 60% similarity. These results suggested a polymorphism distribution of PFGE types of *S.* Enteritidis and *S.* Typhimurium in the Southwest of Shanghai (Figures 5, 6).

#### Annual Similarity of *Salmonella* Enteritidis From 2014 to 2017

The homologous proportion of 12 strains in 2014 was 41.7, 16.7, 16.7, 8.3, 8.3, and 8.3% (F’ type 5/12, D’ type 2/12, E’ type 2/12, A’ type 1/12, H’ type 1/12, J’ type 1/12). The homologous proportion of 7 strains in 2015 was 42.8, 14.3, 28.6, and 14.3% (A’ type 3/7, B’ type 1/7, C’ type 2/7, G’ type 1/7). The homologous proportion of 8 strains in 2016 was 75, 12.5, and 12.5% (A’ type 6/8, I’ type 1/8, J’ type 1/8). The homologous proportion in 2017 was 100% (D’ type 6/6).

### The Annual Similarity of *S.* Typhimurium From 2014 to 2017

The homologous proportion of 12 strains in 2014 was 66.7, 8.3, 8.3, 8.3, and 8.3% (N type 8/12, F type 1/12, J type 1/12, K type 1/12, M type 1/12) and the proportion of 9 strains in 2015 was 44.4, 33.3, and 22.2% (O type 4/9, D type 3/9, L type 2/9). The homologous proportion of 9 strains in 2016 was 75, 12.5, and 12.5% (A type 6/8, C type 1/8, F type 1/8) and the proportion for 31 strains in 2017 29.0, 19.3, 12.9, 12.9, 3.2, 3.2, 3.2, 3.2, 3.2, and 3.2% (C type 9/31, E type 6/31, A type 4/31, K type 3/31, P type 3/31, B type 1/31, G type 1/31, H type 1/31, I type 1/31, J type 1/31, N type 1/31).

Figures 5, 6 show varying degrees of similarity among *S.* Enteritidis and *S*. Typhimurium strains collected each year. In addition, the figures show that in the same year, there was an epidemic of the same strain in Southwest Shanghai. This phenomenon can probably be attributed to the rich seafood resources in Southwest Shanghai, which is located close to the East China Sea. In terms of the inspection time, most samples were sent for laboratory analysis between May and September, which implies that the prevalence of a particular strain may be related to local seasonal eating habits.

## Discussion

The strain types responsible for bacterial diarrhea have been shown to change over time and according to the district analyzed (Xu and Zhang, 2012; Lamberti et al., 2014). The present study clearly demonstrated that *Vibrio parahaemolyticus* was the dominant infectious strain in the Southwest of Shanghai between 1998 and 2007. After 2007, there was a remarkable increase in *Salmonella* as the dominant infectious strain. Our findings suggest that infectious factors for diarrhea have undergone great changes in the Shanghai region over the last decade. Due to improvements in the sanitary management of water resources in 2007, especially in rural areas of Shanghai, the rate of water infection has clearly decreased, with dietary infection becoming the main cause of infectious diarrhea (Shen, 2008).

From 2006, Shanghai residents have participated in the global testing program for non-typhoid *Salmonella* led by the WHO (Zhang et al., 2014). *S.* Typhimurium was found to be the dominant strain responsible for non-typhoid *Salmonella* infectious diarrhea in Shanghai (Zhang et al., 2014). In our study, *S.* Typhimurium accounted for 21.59% of the total *Salmonella* infections from 1998 to 2017, followed by *S*. Enteritidis (16.81%). PFGE analysis was used to characterize 330 *Salmonella* strains from 421 strains collected between 2014 and 2017. The results demonstrated that 33 *S.* Enteritidis strains could be classified into 9 PFGE types, and 60 *S*. Typhimurium organisms into 16 types. The results showed that *S*. Typhimurium was the dominant group in recent years in Shanghai, followed by *S.* Enteritidis, which presented with a polymorphism distribution. In addition, *Salmonella enterica* serovar Typhi was often found in blood culture specimens, while *S.* Enteritidis was mostly isolated from feces samples, and *S.* Typhimurium was widely present in urine (Kwambana-Adams et al., 2015). Our study used feces from diarrhea patients as the main specimen.

Annual and monthly distributions of *Salmonella* infections (Figure 2) revealed that they occurred throughout the year, frequently between April and November, but were much more prevalent between May and September, findings in agreement with a previous survey and related published papers (Qi et al., 2008; Lin et al., 2010; Cao et al., 2012). However, the number of patients and the bacterial isolation rate during the last 2 years has decreased according to the annual infectious diarrhea survey. This decrease is probably related to the strengthened supervision of food hygiene in ChinaDaily.com. (2017). However, it is noteworthy that the detected number of *Salmonella* infections during the last 4 years has significantly increased, which might be related to an alteration in the diet of individuals (Matsuoka et al., 2004).

Major suspicious diet history for *Salmonella* infection in this study included ice milk, ice watermelon, seafood and meat. It has also been reported (Centers for Disease Control and Prevention [CDC], 2008; Raguenaud et al., 2012; Smith et al., 2012; Zielicka-Hardy et al., 2012) that the main cause of infectious diarrhea is the consumption of eggs and meat contaminated with *Salmonella*. The findings suggest that *Salmonella* infection is closely associated with food contamination.

In addition, the clinical symptoms of patients with *Salmonella* infection in the present study concurs with previously published research (Qu et al., 2011; Shen et al., 2014a, b), and our study has also shown that *Vibrio parahaemolyticus* infection has decreased, but that *Salmonella* has become the major strain associated with diarrhea since 2011 in Shanghai (Liu et al., 2004, 2008; Chen et al., 2010).

Serious foodborne *Salmonella* outbreaks have been reported many times in the past. Hence, we investigated the antibiotic susceptibility of various pathogens during the last 4 years, with the results revealing that the sensitivity rate of *Salmonella* to commonly used antibiotics was >70%. It is noteworthy that the sensitivity rate to quinolones was decreased compared with a previous study (Qi et al., 2016), so it will be important to continue monitoring the sensitivity of *Salmonella* to these agents. Also, antibiotics should be employed to monitor susceptibility and perhaps prophylactically to prevent the outbreak of serious diarrhea symptoms in patients.

Currently, third-generation cephalosporins and quinolones are the most common antibiotics used to treat *Salmonella* infections in the clinic. With increasing usage and the frequency of prescriptions (Xu and Zhang, 2012; Lamberti et al., 2014), unfortunately many *Salmonella* strains have now become resistant to quinolones, which makes treatment of infection much more difficult and costly. A recent report has shown that about 22.5% of non-typhoid *Salmonella* strains are resistant to at least one antibiotic. The most common multi-antibiotic-resistant phenotypes are to ampicillin, chloramphenicol, streptomycin, sulfonamides and tetracycline (9.35%) (Liu et al., 2004). Patients with diarrhea take non-standard broad-spectrum antibiotics (nalidixic acid, ampicillin, sulfamethoxazole, streptomycin and tetracycline) but resistance is clearly adversely affecting the control and treatment of *Salmonella* infections.

Our study unfortunately did not analyze the antibiotic resistance of the same or different PFGE type strains. However, for identical PFGE strains, they had very similar antibiotic-resistant profiles. For those strains sharing the same PFGE type but different antibiotic-resistant profiles, the antibiotics resistance gene might mutate and also the mutation site is not located at the PFGE restriction enzyme cutting site. As a result, antibiotic resistance cannot be reflected fully in the PFGE classification (Lin et al., 2010). Therefore, the PFGE method can be used as a sensitive method to study genotyping in the molecular epidemiology of *Salmonella* infection. Furthermore, it can help to establish the relationships among cases. Clearly, further research will be necessary to characterize the resistant strains identified by the PFGE method. Nevertheless, the PFGE method can be used clinically to identify the pathogen and to investigate the origin of the outbreak of *Salmonella* poisoning.

A limitation of the present study is that all samples came from a single hospital and therefore our findings may not reflect the situation in other regions of China.

## Conclusion

The proportion of gastrointestinal infectious diarrhea cases caused by *Salmonella* has increased in the Southwest of Shanghai. The types of *Salmonella* bacteria are numerous and are widely distributed. Our study provides a basis for the early clinical identification and diagnosis of pathogens causing intestinal infectious diseases.

## Author Contributions

XQ, XX, SB, and DL conceived and design the study. All authors were responsible for acquisition and analysis of data, commented on the draft, and approved the final version of the manuscript. XQ and DL were in charge of statistical analysis. XQ, PL, SB, and DL drafted the manuscript.

## Conflict of Interest Statement

The authors declare that the research was conducted in the absence of any commercial or financial relationships that could be construed as a potential conflict of interest.
