# Supplementary material for: Epidemiological and Molecular Investigations on Salmonella Responsible for Gastrointestinal Infections in the Southwest of Shanghai From 1998 to 2017
Source: Front Microbiol. 2019 Sep 18;10:2025. doi: 10.3389/fmicb.2019.02025 (PMC6759537; doi:10.3389/fmicb.2019.02025)
Supplement: Supplementary file 2 [file Table_2.DOCX]

**Supplementary Table 1.** **The distribution of pathogens responsible for intestinal infections in the Southwest of Shanghai**

| Year | N | *Salmonella* (565 strains) | *Vibrio parahaemolyticus* (2,489 strains) | *Shigella* (125 strains) |
| --- | --- | --- | --- | --- |
|  |  | Number of strains (n%) | Number of strains (n%) | Number of strains (n%) |
| 1998 | 254 | 2 (0.8) | 239 (94.1) | 13 (5.1) |
| 1999 | 205 | 1 (0.5) | 204 (99.5) | 0 (0) |
| 2000 | 217 | 2 (0.9) | 205 (94.5) | 10 (4.6) |
| 2001 | 436 | 2 (0.5) | 420 (96.3) | 14 (3.2) |
| 2002 | 422 | 1 (0.2) | 392 (92.9) | 26 (6.2) |
| 2003 | 397 | 0 (0) | 393 (99) | 4 (1) |
| 2004 | 221 | 1 (0.5) | 207 (93.7) | 12 (5.4) |
| 2005 | 110 | 0 (0) | 97 (88.2) | 13 (11.8) |
| 2006 | 59 | 8 (6.8) | 51 (86.4) | 4 (6.8) |
| 2007 | 59 | 18 (27.1) | 39 (66.1) | 3 (5.1) |
| 2008 | 79 | 33 (37.97) | 47 (59.49) | 2 (2.53) |
| 2009 | 77 | 26 (33.37) | 51 (66.23) | 0 (0) |
| 2010 | 130 | 30 (23.08) | 89 (68.46) | 11 (8.46) |
| 2011 | 84 | 51 (60.71) | 20 (23.81) | 13 (15.48) |
| 2012 | 31 | 26 (83.87) | 5 (16.13) | 0 (0) |
| 2013 | 64 | 34 (53.13) | 30 (46.88) | 0 (0) |
| 2014 | 77 | 58 (75.32) | 19 (24.68) | 0 (0) |
| 2015 | 65 | 50 (76.92) | 15 (23.08) | 0 (0) |
| 2016 | 120 | 102 (85) | 18 (15) | 0 (0) |
| 2017 | 159 | 120 (75.47) | 39 (24.53) | 0 (0) |
| Total | 3,179 | 565 (17.77) | 2489 (78.3) | 125 (3.93) |
